# Temporal interpretation of right ventricular strain after pulmonary embolism

**DOI:** 10.1093/ehjimp/qyag174

**Published:** 2026-10-07

**Authors:** Hasan Burak Isleyen, Sercan Bulut

**Affiliations:** Department of Cardiology, Istanbul Nisantasi University Faculty of Medicine, Istanbul 34398, Türkiye; Department of Cardiology, Bezmialem Vakif University Faculty of Medicine Hospital, Istanbul 34093, Türkiye; Cardiology Clinic, Bağcılar Training and Research Hospital, Istanbul, Türkiye

Farmakis and colleagues assessed right ventricular (RV) strain in 175 survivors of acute pulmonary embolism (PE).^1^ Sixteen participants developed post-PE impairment (PPEI) or chronic thromboembolic pulmonary hypertension (CTEPH) during follow-up. Three-month RV global longitudinal strain was associated with the combined outcome over 2 years (odds ratio 1.10, 95% confidence interval 1.01–1.21).^1^ The timing of outcome onset relative to the 3-month scan determines the clinical interpretation of this association.

In the parent FOCUS study, PPEI was a combination of persistent or worsening clinical, functional, biochemical, and imaging abnormalities assessed during follow-up.^2^ Some participants may therefore have met PPEI criteria by the time of the three-month echocardiogram. CTEPH can likewise be recognized during early follow-up.^2^ If the outcome includes abnormalities already present at the index scan, the strain measurement may characterize concurrent impairment rather than predict new disease.

The most informative distinction would be the number of participants who had already fulfilled PPEI or CTEPH criteria at the 3-month examination and the number who first met the criteria thereafter. Among those free of the outcome at 3 months, an association between 3-month RV strain and subsequent incident outcomes would support prognostic interpretation. If the small number of later events precludes a stable estimate, a descriptive separation of prevalent and incident events would still clarify the proposed clinical role.

This distinction also matters for the combined endpoint. An association driven primarily by impairment detected at the same visit would support RV strain as a contemporaneous assessment marker. An association with later CTEPH or PPEI would provide different evidence for follow-up risk stratification. We encourage the authors to distinguish these interpretations when describing 3-month RV strain as an early marker of late complications.

## Data Availability

No new data were generated or analysed.

## Lead author biography

Asst. Prof. Hasan Burak Isleyen, MD, is an Assistant Professor of Cardiology at Istanbul Nisantasi University Faculty of Medicine in Istanbul, Türkiye.
